# Foreign Items

**Published:** 1869-11

**Authors:** 


### Foreign Items.

On the 1st of September, during the discussion upon vaccination, which has occupied the attention of the Imperial Academy of Medicine (Paris) during a portion of every session, for several months past, M. Ricord made the following observations: "There are now under discussion two species, or rather two sources of vaccine, with but one and the same virus. The first, which we have from Jenner, is asserted to be degenerated. MM. Guérin, Bousquet, and Vernois have treated this question too thoroughly to permit its recall. I do not believe in the degeneration of vaccine; I attribute the differences observed to the soil, rather than to the grain. But another stigma is affixed to human vaccine. If, they say, it has not degenerated, it has become vitiated; it has become compromised by being united with another virus; in a word, they present to us vaccinal-syphilis. The mode of transmission of syphilis has been a long time denied and neglected by the Academy itself. I ask of M. Depaul, who has made an immense number of vaccinations, if he took all the minute precautions which would avail to prevent such an accident? Certainly not; he did not so occupy himself, he did not think of them; his reports do not contain a single word upon this subject, previous to what might be called the Italian Epidemic.

"To sum up, then, vaccinal-syphilis is very rare, excessively rare, very difficult to reproduce, even when the inoculation is performed with vaccine from subjects notoriously syphilitic.

"Moreover, at one time, nothing was talked of but vaccinal-syphilis; facts of this sort were multiplied infinitely; following these indications, it might have been supposed that vaccinal-syphilis would soon replace vaccine. This has been considerably exaggerated. A large number of observations have aggregated which presented no traces of syphilis. Moreover, M. Depaul himself has told you, when we begin to investigate a subject we take whatever we can, subsequently we reject a portion of these doubtful facts, which had previously been accepted.

"Like M. Guérin, I am astonished at one thing. I cannot restrain my surprise at the great number of children cured without treatment, after having had this pseudo vaccinal-syphilis.

"We know, concerning infantile syphilis, however great its gravity, what extreme difficulties we experience in curing it by specific treatment carefully managed, and long continued. I know that it has been said, 'We must distinguish between congenital and inoculated infantile syphilis: the first is much more grave, because it seizes the infant before it has sufficient vigor to resist.' So they say; but is syphilis inoculated into infants benignant? Certainly not, as much as it might be. I can assert that it is severe, very severe, altogether as grave, at the least, as any other sort. It is said further that syphilis, inoculated, should be weaker in consequence of the inoculation; but when it is taken by contact at its habitual source, it is inoculated; we must not delude ourselves upon this point. An old author, Boerhave, I think, has expressed the contrary opinion to that which is at present held; according to him, syphilis would be much more severe, were it not acquired in the habitual way.

"Permit me to recall to you, that when I did not believe in the contagion of secondary lesions, when the effort was made to prove this against me and to combat my opinions, all the cases of syphilis produced by inoculation of secondary lesions were of the most severe character, nurses infected by their nurselings were lost women (femmes perdues); now the point of observation has been changed, and these same facts have become most benign. Let us, then, acknowledge the truth, that in children, as in adults, syphilis may be mild or severe, but that whether inoculated or not, it is always less easy to cure in young infants, because they offer less resistance. In my own mind, this is a well established conviction, based upon long practical experience.

"I do not admit the pretended benignity of infantile syphilis.

"M. Guérin, on his part, has, perhaps, exaggerated a little in the other direction. He has nearly reached the conclusion that the diagnosis of syphilis was very vague and uncertain. I know nothing, on the contrary, in medicine or surgery, more easy to diagnose than syphilis, in the vast majority of cases; primary phenomena, indurated chancre, then adenopathia and secondary and tertiary lesions more or less remote; when this picture is presented to the view, it is difficult to be deceived. I know very well, that it may happen that we may be satisfied with insufficient evidence; here, even, in the Academy cases have been presented which were deceptive from too great precipitation. But we are rarely deceived, deliberately. Thus I myself have been forced to admit the reality of vaccinal-syphilis, in spite of all my repugnance. Facts have constrained me thereto. They have been presented to me with characteristics which did not admit of doubt, for I described them myself. I decided that I was deceived, and I acknowledged it. I know nothing more agreeable than to recognize an error which has been demonstrated by one's own rules. I therefore now admit the possible transmission of syphilis by vaccination. But I must say it, it is something which has thus far escaped me. I have not yet been able to discover in published observations, the first '*vaccino-siphiliphere*.'

"In those of the 2nd or 3rd hand, that is alone sufficient, for inoculation produces a chancre, which itself becomes a source of syphilis. But can the first, which was itself the subject of constitutional syphilis, develop in itself any syphilitic lesion at the vaccinated point? It is a curious fact, that upon those who are constitutionally syphilitic, all possible operations may be performed without any modification of their results by syphilis. For my own part I have never seen, in my long practice, that it exercised the least influence. I have removed tonsils covered with mucous tubercles, performed amputations, castrations, etc., without finding the cures retarded or the cicatrization less perfect than in non-syphilitic subjects. I have never seen a vaccinal pustule become chancrous.

"M. Depaul—It never becomes such.

"M. Ricord—I am happy to be, upon this point, of the same opinion as M. Depaul.

"It has been said, and it appears just at the first glance, is it necessary to know the source from which a disease is drawn, in order to recognize it? No, doubtless; and it happens to me every day to recognize the pox without knowing from whence it is derived. But is it, then, absolutely a matter of indifference to trace it to its source? By no means; frequently, it would be very interesting to study it. I should like very much to study the vaccinal pustule which supplies the virus contaminated by syphilis.

"Vaccinal-syphilis admitted, and I repeat it, I do admit it, what is its mechanism? What are its pathogenic conditions? Is there a union of the two species of virus, with a hybrid product? Certainly not. Are they then placed parallel? It is possible. There is one point upon which I insist; the question of the blood and its agency in the transmission of syphilis. I believe it impossible for the most adroit to avoid the blood in collecting vaccine upon his lancet. I have asked, from the Academy, the purest possible vaccine virus, with special reference to this point. I have submitted it to M. Robin; it contained numerous globules of blood.

"M. Depaul—It is always so under the microscope; blood globules are always detected in the purest vaccine.

"I am happy to be again confirmed in my opinion by the authority of M. Depaul. Thus when they picture for us pure vaccine in the vaccinal pustule, and syphilis in the sub-soil, if the expression may be permitted, they invoke theoretical subtleties based upon nothing at all, and which, moreover, possess the serious inconvenience of endangering the vaccinator, if syphilis should manifest itself in the vaccinated child. It may be believed that he has not taken the necessary precautions, or again that he has taken them all; that he has neglected to avoid blood; then that it was impossible to avoid it. It must be said, if syphilis can come from a vaccine pustule, it may be derived from the vaccine as well as from the blood.

"I now come to cow-pox. Its panegyric has been too recently pronounced by M. Depaul to admit of its repetition. M. Herard, moreover, has traced between it and the Jennerian virus, a relation which has shown with sufficient clearness their possession of equal qualities. Here, at least, there is no fear of syphilis, for the animal is exempt. I was one of a committee, which made every possible effort to inoculate syphilis into the cow, without success. This is certainly an advantage. But may not the cow-pox at some time have its contamination? Animals have diseases which are communicated to men.

"To conclude, vaccinal syphilis is rare. Neither human vaccine nor the cow-pox should be rejected. They should both be investigated, and at the same time propagated.

